# Remote Patient Monitoring System for Polypathological Older Adults at High Risk for Hospitalization: Retrospective Cohort Study

**DOI:** 10.2196/71527

**Published:** 2025-07-14

**Authors:** Damien Testa, Israa Salma, Vincent Iborra, Victoire Roussel, Mireille Dutech, Etienne Minvielle, Elise Cabanes

**Affiliations:** 1 EPOCA U&I, 1-3 Allée du Tertre, Nanterre, 92500, France, 33 1 79 35 59 74; 2 Institut Polytechnique de Paris, Centre de Recherche en Gestion de l’Ecole Polytechnique (I3-CRG, UMR CNRS 9217), Palaiseau, France; 3 Gustave Roussy Cancer Centre, Département Interdisciplinaire d’Organisation du Parcours Patient (DIOPP), Villejuif, France

**Keywords:** geriatric care, telemonitoring, chronically ill patients, health care outcomes, unplanned hospitalization, emergency department visits, general practitioner

## Abstract

**Background:**

Health care systems are increasingly facing challenges posed by the aging of populations. In particular, hospitalization, both initial and subsequent, is often observed among older adult patients. However, research suggests that nearly 23% of all hospitalizations could be avoided. In this perspective, remote patient monitoring (RPM) systems are emerging as a promising solution, enabling professionals to detect and manage patient complexities early within home-based care settings.

**Objective:**

This study aims to provide additional analyses regarding the impact of the EPOCA RPM system for polypathological older adult patients on the total number of unplanned hospitalization days and admissions, as well as emergency department (ED) visits. In a prior study, we evaluated the impact when the operator of the RPM system is a geriatrician. In this study, we assess the impact when the general practitioner is the operator.

**Methods:**

We used a retrospective, before-and-after cohort design. Polypathological older adult patients aged 70 and older, who benefited from the EPOCA RPM system for at least 1 year (between February 2022 and August 2024), were included in the analysis. We compared the outcomes between the previous year (Y–1) and the follow-up year (Y) by the EPOCA RPM system. Statistical analyses were significant at *P* value <.05

**Results:**

In total, 80 patients were included in the analysis, with an average age of 87. The results showed a significant reduction (*P*<.001) between Y–1 and Y in the total number of unplanned hospital admissions (by 57%), hospitalization days (by 49%), and ED visits (by 62%). Our findings reflected a significant decrease per patient from 0.99 to 0.42 in hospital admissions, from 0.99 to 0.37 in ED visits, and a reduction of 9.7 hospitalization days per year (*P*<.001). Additional analyses stratifying by hospitalization history, disability level, and caregiver status showed that the greatest effect of the RPM system was on patients with high risk and severe disability. Finally, there was no observed increase in mortality or transfers to intensive care units.

**Conclusions:**

Our findings are consistent with our previous results regarding the potential benefits of the EPOCA RPM system in managing care for polypathological older adult patients, this time with general practitioners as system operators. They also support existing evidence on the promise of RPM in improving care and health outcomes for older adult patients while alleviating hospital burdens by reducing unplanned hospitalizations and ED visits. It is, therefore, essential to incorporate reimbursement policies for these RPM initiatives so as to facilitate their adoption within health care systems and enhance their impact on health outcomes.

## Introduction

The rapid aging of populations in Europe and worldwide in recent decades is posing major social and economic challenges, putting increasing pressure on already overburdened and costly health care systems [1]. Older individuals often experience difficulties from multiple chronic conditions, resulting in growing demands for complex health care services and specialized treatments along their care pathways, from hospital to ambulatory care settings [2].

In France, by 2024, 28% of the population (18.5 million) was aged 60 years or older [3], and nearly 6.1% were aged 80 years or older (around 4 million) [4]. These demographic shifts are placing enormous pressure on France’s health care infrastructures, which are already facing shortages of geriatricians and a declining workforce of general practitioners (GPs). According to the latest available data, in 2022, only 2418 geriatricians were working in older adults care, most of them in nursing homes and hospitals [5].

The result, therefore, is a fragmented care system leading to reactivity, characterized by an increased rate of initial and subsequent hospitalizations for older adult patients [6], often considered as the primary response to manage complexities associated with their chronic conditions. However, such a hospital-based care approach poses major organizational challenges, particularly in emergency departments (EDs) [2]. French statistics from 2018 indicated that 12.5% of hospital admissions concerned patients aged 80 years and older [7], 40% of them experienced at least 1 hospital stay per year, with an average duration of 25 days. However, estimates from previous research suggest that approximately 23% of all hospital admissions could be avoided [8]. The prevalence of the inappropriate use of EDs (ie, urgent problems that could be managed in a primary care setting) also ranges from 20% to 40% and is often associated with age and income variables [9,10]. Reducing avoidable hospitalizations and unnecessary ED visits can have several benefits, including improved patient outcomes and optimized health care resource use. Most importantly, it prevents adverse events associated with hospital stays, such as functional and cognitive impairments, especially among older adult patients [11-13]. By avoiding unnecessary hospitalization, older adults can also benefit from maintaining their autonomy level through self-management of their chronic conditions while preventing transitions to long-term care facilities. These benefits may result in improved quality of life [14,15], overall quality of care, and increased patient satisfaction [16]. From another perspective, reducing unnecessary hospitalizations can also be advantageous in reducing health care expenditures and the economic burden while enhancing hospital resource allocation, such as bed capacity [15,16]. These observations underscore the need for primary care-based solutions that can be highly responsive and accessible, designed to meet and manage patients’ changing needs. Especially those with complex and chronic conditions and at risk for deterioration and potentially hospitalization, such as polypathological older adults.

In this context, remote patient monitoring (RPM) systems emerge as a promising solution for enhancing the quality of care provided to chronically ill patients within home-based settings. This approach, also known as telemonitoring, consists of a form of telemedicine that enables health care professionals to remotely track patients’ health status and take actions when abnormalities occur. Such an approach facilitates the sharing and coordination of resources [17]. It also allows real-time health information to be exchanged between patients and care providers through digital technologies [18]. The major benefit of this approach compared to other traditional care management strategies, such as in-home nursing care and regular physicians’ check-ups, lies in improving health care accessibility. By offering continuous 24/7 monitoring, it enables early detection of health deterioration, facilitates remote care management, and allows rapid intervention in emergencies [19,20]. RPM systems hold great potential to help prevent critical health conditions for older adult patients. It can thus reduce avoidable hospitalizations as well as other health care resource utilization [21-23]. Previous reviews showed that some RPM solutions seem effective in specific clinical settings, and their benefits can vary across different contexts and remain inconclusive [24,25]. It is therefore essential to provide further evidence on RPM systems to fully understand their impact on health care outcomes for complex older adult patients.

From this perspective, this paper provides additional evidence on RPM systems’ impact on health care outcomes for older adult patients in the French context. It presents a complementary analysis evaluating the impact of the EPOCA RPM system on health care utilization, particularly in terms of unplanned hospital admissions, ED visits, and the length of hospital stays for polypathological older adult individuals. In a previous study, we reported findings from the implementation of the system when operated by a geriatrician [26] as part of the French Social Security Financing Act (Vigie-Âge, Article 51 experiment) [27]. However, in the context of limited geriatricians and the increasing number of patients, it is necessary to set up telemonitoring with local health care professionals in which GPs play a critical role. Therefore, in this study, we present the results of the EPOCA RPM system when operated by the GP.

## Methods

### Study Design

This study uses a retrospective, before-and-after cohort design involving patients who benefited from the EPOCA RPM system between February 2022 and August 2024.

### Inclusion Criteria

Participants aged 70 years or older with polypathology (ie, having multiple chronic diseases, 2 or more affected systems) and who have been part of the long-term care pathway within EPOCA (at least 1 year) were included in the analysis.

### Intervention

The EPOCA RPM system [28] is a telemedicine solution offering secure home support for chronically ill older adults. It includes a 24/7 RPM platform for continuous remote medical monitoring and emergency assistance. The system facilitates coordination between care providers, GPs, and geriatricians to create personalized care plans. It consists of 2 main elements: a connected device (such as a wristband) for transmitting health data and an RPM center staffed by qualified and trained nurses and medical practitioners who can communicate with both patients and caregivers when needed. Every patient enrolled in the RPM system receives an initial home visit, during which their needs and health status are thoroughly assessed. They also receive teleconsultations and personalized installation of remote monitoring devices, as well as training and advice on the use of these devices. Further details about the EPOCA intervention can be found in our previous study, in which the geriatrician is the operator of remote care [26].

### Outcomes

The outcome measures for this study were the total number of unplanned hospital admissions, including ED visits, the hospitalization rate per patient, and the cumulative length of hospital stay in days for both the total population and per patient. Outcomes were compared between the year preceding the patient’s enrollment in the RPM system (Y–1) and after 1 year of follow-up (Y).

### Data Collection

Patient data are collected by medical and paramedical teams and compiled in the EPOCA RPM database at the inclusion and throughout the follow-up period. Data related to the study objectives for patients included are extracted from the EPOCA database for (Y–1) and the follow-up year (Y). To minimize potential bias in Y–1 data collection, a systematic and thorough approach is adopted when including patients in the RPM program, using medical records and direct communication with the patient’s care team. All data were anonymized before extraction and analysis. All analyses were conducted in a secure HDS (health data hosting) space.

### Statistical Analysis

Descriptive statistics were used to provide detailed information about the study cohort. Continuous variables (ie, sociodemographic and clinical data) were expressed by mean and SD. Categorical variables were reported as numbers and percentages.

We first used a Shapiro-Wilk test to assess the normality of the data distribution. We then applied the Wilcoxon test, a nonparametric approach, to compare outcomes in the year before (Y–1) and after (Y) the intervention. Statistical significance was defined at *P*<.05. All statistical analyses were performed using Visual Studio CodeTM (Microsoft), version 1.84.2, in Python language (version 3.11.5). No data imputation was required for the statistical analysis since all outcome data were fully available for comparison between the baseline year (Y–1) and the follow-up year (Y).

A generalized linear model with a negative binomial distribution and a log link function was used to account for overdispersion in the count outcome. Model selection was based on the theoretical relevance and statistical significance of predictors.

### Ethical Considerations

This study follows the MR004 framework of French research methodology; it requires a CNIL (Commission Nationale de l’Informatique et des Libertés) declaration and patient consent. However, ethics committee approval—equivalent to institutional review board approval—was not required, given the retrospective nature of the data analysis. In this context, the minimum dataset was declared to the French authorities under CNIL authorization number 2234081, in accordance with the General Data Protection Regulation (GDPR). All patients enrolled in the EPOCA RPM system systematically receive written information indicating that data collected from the EPOCA RPM database system, including medical records and follow-up outcomes, could be used retrospectively for research purposes. They also provide written consent for the use of their anonymized data following their enrollment in the EPOCA RPM system. No compensation was provided to participants.

## Results

### Patient Characteristics

**Figure 1.**
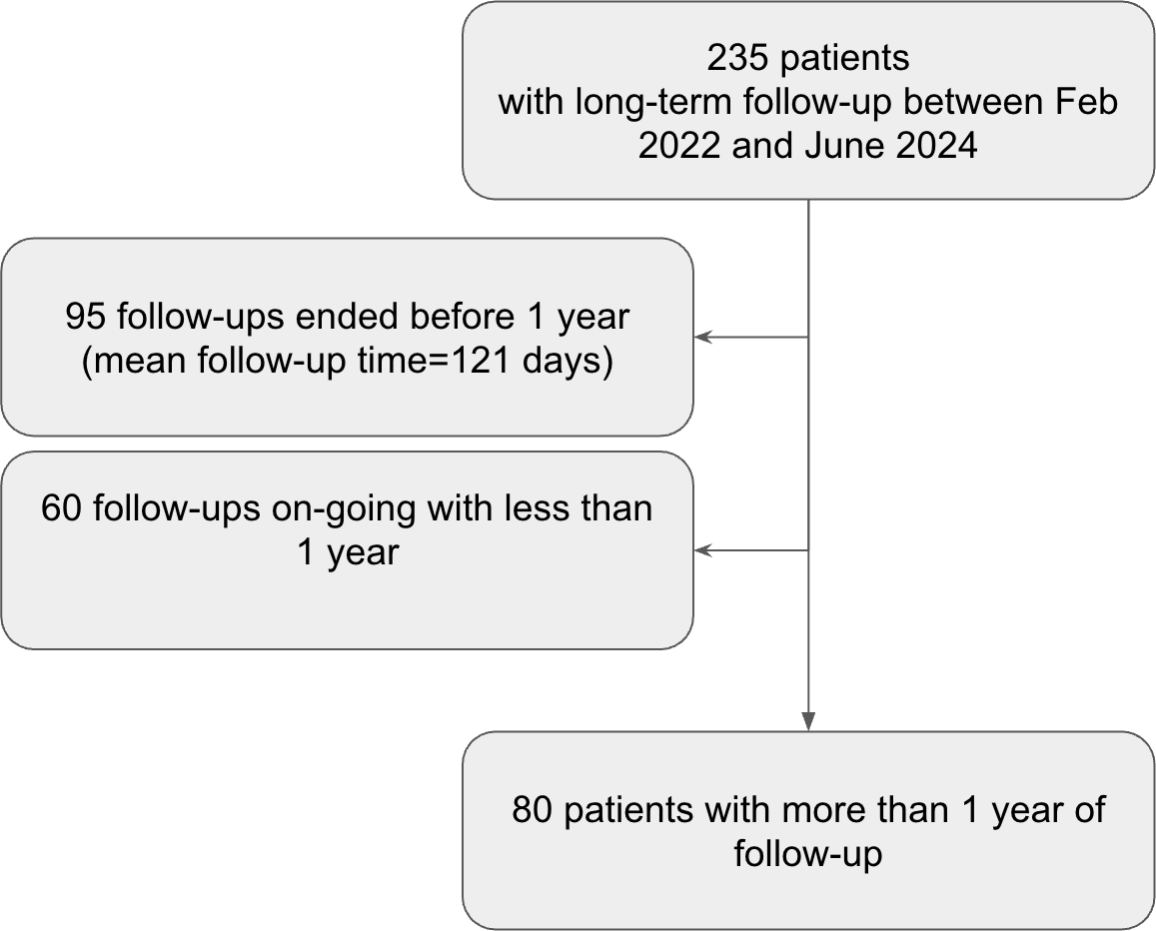
Flow chart of participant selection from the total number of patients included.

Of the 235 eligible study participants, only 80 met the inclusion criteria (ie, aged 70 years or older, with polypathology and more than 1 year of follow-up) and were included in the analyses (see Figure 1). Patients’ characteristics are presented in Table 1. The mean age of participants was 86.7 (SD 8.3) years, and the mean of comorbidities was 6.7 (SD 3.7). Females represented 69% (n=55) of the sample size. Approximately 68% (n=54) of participants were assisted by a nonprofessional caregiver, and 55% (n=44) were living alone at home. The most prevalent referrals for the intervention were from nurses by 49% (n=39), followed by hospitals by 22% (n=18), EDs by 20% (n=16), long-stay units by 5% (n=4), and GPs by 4% (n=3). Further details on patient characteristics are provided in Multimedia Appendix 1.

**Table 1.** Characteristics of patients included in the study.

Variables	Patients
Demographic characteristics (N=80)	
Age (years), mean (SD)	86.7 (8.3)
Sex (female), n (%)	55 (69)
Presence of caregiver (N=80) n (%)	
Nonprofessional caregiver, n (%)	54 (68)
Professional caregivers, mean (SD)	2.4 (1.7)
Functional and cognitive assessment	
GIR^a^ (n=73), mean (SD)	3.6 (1.5)
ADLs^b^—Katz index (n=68), mean (SD)	4.0 (1.9)
IADL^c^—Lawton index (n=62), mean (SD)	3.8 (2.7)
Comorbidities (n=80)	
Number of chronic diseases, mean (SD)	6.7 (3.7)

a GIR: Groupe Iso-Ressources (Iso-resources group).

b ADL: activities of daily living.

c IADL: Instrumental activity of daily living.

### Evaluation Outcomes

Overall, the results of our study showed a significant improvement in all outcome measures between Y–1 and the follow-up year (Y) by the EPOCA RPM system. This was observed with significant reductions in health care use, detailed in Table 2. The overall number of unplanned hospital days dropped by 49%, which resulted in an average reduction of 9.7 days spent in the hospital per patient each year. The number of unplanned hospital admissions decreased by more than half (57%), while emergency room visits declined by two-thirds (62%). During the Y–1 period, 60 (75%) of individuals required hospitalization at least once. This number dropped to 21 (26%) over the follow-up period (Y).

**Table 2.** Outcome measures: a Y–1^a^ versus Y^b^ analysis.

Outcome measures, total participants (N=80)	Y–1 period		Y period		RRR^c^	
	n/mean (SD)	95% CI	n/mean (SD)	95%CI	% (95% CI)	*P* value
Unplanned hospitalization duration (in days)	1575/19.7 (31.2)	13.4‐26.8	799/9.99 (31.6)	3.8‐17.6	49 (31.3‐85.8)	<.001
Unplanned hospitalizations	79/0.99 (0.75)	0.83‐1.15	34/0.42 (0.84)	0.26‐0.61	57 (26.5‐77.4)	<.001
ED^d^ visits	79/0.99 (0.85)	0.80‐1.18	30/0.37 (0.72)	0.22‐0.54	62 (32.5‐81.4)	<.001

a Y–1: year preceding the patient’s enrollment in the system.

b Y: follow-up year.

c RRR (%)/*P* value: relative risk reduction refers to the proportional decrease in risk of event between the Y–1 and Y and statistical significance, respectively.

d ED: emergency department.

To identify predictor factors associated with the number of hospitalization days in the follow-up period (Y), we conducted a secondary analysis using a generalized linear model. The results, presented in Table 3, identified several variables significantly linked to hospitalization duration, *R*²=0.84. For instance, compared to females , males were linked to a significantly lower number of hospital days (*β*=−3.862, *P*<.001). Similarly, greater autonomy, which can be seen through higher GIR (Groupe Iso-Ressources (iso-resources group)) scores (*β*=−.827, *P*<.001), and an increased number of hospitalization days in the Y–1 (*β*=−.037, *P*<.001) were both associated with shorter stays. Interestingly, the increased number of comorbidities was also a variable significantly linked to fewer hospitalization days (*β*=−.203, *P*<.001). In contrast, other variables such as age, functional status (Activities of Daily Living), and the number of professional caregivers did not show a significant association with the hospitalization days.

**Table 3.** Factors associated with the number of hospitalization days in the Y^a^ period (n=80). Model: generalized linear model analysis results.

Factors	Coefficient (β)	SE^b^	Z value	*P* value	95% CI^c^
Intercept	10.393	2.486	4.180	<.001	5.520, 15.267
Sex (male)	−3.862	0.547	−7.058	<.001	−4.935, −2.790
ADLs^d^	−0.159	0.136	−1.166	.244	−0.425, 0.108
Age	−0.026	0.024	−1.077	.281	−0.072, 0.021
GIR^e^	−0.827	0.186	−4.445	<.001	−1.191, −0.462
Number of chronic diseases	−0.203	0.046	−4.426	<.001	−0.293, −0.113
Number of professional caregivers	−0.132	0.114	−1.164	.244	−0.355, 0.090
Number of hospitalization days in Y–1^f^ period	−0.037	0.009	−4.313	<.001	−0.054, −0.020

a Y: follow-up year.

b SE: standard error.

c 95 % CI: Confidence interval. Values represent the 95% CI of the β coefficient.

d ADL: Activities of Daily Living

e GIR: Groupe Iso-Ressources (iso-ressources group).

f Y-1: year preceding the patient’s enrollment in the system.

To further explore whether the impact of the RPM system varied based on patient characteristics, we conducted additional stratified analyses on the study’s main outcomes. These analyses were based on stratification by (1) the number of hospitalizations in the period Y-1 (Y–1: 0, 1, or 2+ hospitalizations), (2) the disability level using the GIR score (severe disability GIR ≤3 and no or moderate disability GIR ≥4), and (3) the presence or absence of a nonprofessional caregiver. The results are presented in Multimedia Appendix 2

Overall, the stratified analysis showed significant relative risk reduction across all groups, regardless of patient characteristics. Interestingly, when stratifying by hospitalization history, patients with 2 or more hospitalizations in the Y–1 period experienced significantly greater reductions across all outcomes compared to patients with 1 or no prior hospitalizations. Similarly, patients with severe disability (GIR ≤3) showed greater relative reductions compared to those with no or moderate disability. Finally, when we stratified by the nonprofessional caregiver status, both groups revealed significant reductions.

## Discussion

### Principal Findings

This retrospective study involved a before-and-after comparison of health care resource utilization among 80 polypathological older adult patients, who were followed for at least 1 year with the EPOCA RPM system when the GP was the system operator. Compared with the previous year (Y–1), a significant reduction, by half, was observed in the total number of unplanned hospitalization days and admissions, as well as ED visits. The main contribution of this study lies in providing additional evidence supporting RPM systems’ potentials in improving health outcomes and optimizing care delivery for older adult patients with complex chronic conditions.

### Comparison With Prior Findings: The Geriatrician as System Operator

Our results are similar to those of a before-and-after analysis carried out on 120 polypathological older adult patients (6.1 comorbidities, mean age 86.8; further details are presented in Multimedia Appendix 3), followed by the EPOCA RPM system, and when the system operator was the hospital geriatrician [26]. A reduction of 63%, 48%, and 48% was also observed in the total number of unplanned hospitalization days, admissions, and ED visits. This comparability of results suggests 2 main implications. First, the effectiveness of the EPOCA RPM system, as it is associated with a consistent improvement in patient outcomes across different settings in the French context. This was seen through a reduced use of health care resources, in particular the rate and duration of unplanned hospitalizations and ED visits, with 2 different system operators (ie, geriatricians and GPs). However, a large-scale randomized control trial (RCT) is required to confirm the effectiveness of our system compared to a control arm. Second, in the context of a limited number of geriatricians in France [7], such an at-home RPM solution, involving geriatric expertise, could be beneficial for the geriatric population. Particularly when the majority of patients aged 70 years and older have a GP. Only 5.6% of patients aged 70 to 79 and 5.7% of patients aged 80 years and older do not have a GP [29]. Therefore, our study underscores the potential benefits of adopting RPM solutions, not only by geriatricians, but also by GPs and local health care institutions, as a useful strategy to manage complex conditions, prevent clinical deterioration, and thus reduce burdens on the French health care system.

### Comparison With the Literature

Our results also align with previous findings suggesting a positive impact of RPM systems on patient clinical outcomes and overall health care improvement [30,31], particularly in reducing hospitalization risk, as demonstrated in both national and international studies. In previous RCTs, the risk was lowered by 57% [23], 28% [22] in intervention groups compared to conventional care groups, and 33.2% in a before-after cohort [32] (Table 4). However, in terms of ED visits, our findings differ from those of another French RCT, the eCOBAHLT study, which showed an increase in ED visits uncorrelated with any increase in hospitalizations. This increase was attributed to the unavailability of treating physicians, leading patients with manageable health conditions to seek care in the ED [22]. Contextual and methodological challenges may also explain these inconsistencies.

**Table 4.** Comparison of outcome measures: EPOCA RPM^a^ versus international RPM programs.

Study (type)	Unplanned hospitalizations, RRR^b^ (%) (*P* value)	ED^c^ visits, RRR (%) (*P* value)
EPOCA study (retrospective cohort)	57 (<.001)	62 (<.001)
eCOBAHLT study (RCT^d^) [16]	28 (.04)	Increased risk of 4.2 (.03)
CONNECARE study (RCT) [17]	57 (.001)	56 (.01)
ValCrònic program (quasi-experimental before-after) [26]	33.2 (<.001)	32.2 (<.001)

a RPM: remote patient monitoring.

b RRR (%)/*P* value: relative risk reduction between the Y–1 and Y/and statistical significance.

c ED: emergency department.

d RCT: randomized control trial.

### Interpretation of Complementary Findings and Implications for Future Research

Multiple predictors were significantly associated with reduced hospitalization duration in the follow-up year (Table 3). The strongest predictors include male sex, patient disability level, number of hospitalization days in the Y–1 period, and, interestingly, number of comorbidities. Combining these findings with complementary stratified analyses, the results showed that the greatest effect of the RPM system was observed in patient subgroups with a history of 2 or more hospitalizations, severe disability (GIR score ≤3), or without a nonprofessional caregiver. These observations suggest that high-risk patients may benefit the most from the EPOCA RPM system, likely reflecting its structured and coordinated care model. This interpretation is supported by the fact that the RPM team works closely with external and ambulatory health care professionals (mean of 2.4 professionals per patient) [26]. This approach allows proactive management of patients with complex health conditions, thus stabilizing their health status, preventing complications, and avoiding hospitalizations [33]. It is important to note that, among the subgroups, for patients without hospitalization history in the Y–1 period, the effect of the RPM was difficult to measure. In future research, this variable, history of hospitalization, could be considered as an inclusion criterion to minimize bias and improve the precision of effect estimates. Other variables such as GIR score and caregiver status could be used for more detailed stratified analyses in a large-scale study.

Finally, while the finding of a correlation between the number of comorbidities and a reduced hospitalization duration seems counterintuitive, it can be explained by the specific profile of patients followed by the EPOCA RPM system. These patients are predominantly polypathological, with an average of 6.7 comorbidities. Such complex profiles represent the ideal target population who may benefit the most from close home-based care and personalized health management through RPM systems like EPOCA’s. Looking ahead, further in-depth research on RPM interventions is therefore essential to fully understand and measure its impact on care systems and health outcomes of polypathological older adult patients.

### Risk and Sustainability Challenges of RPM Interventions: Policy Recommendations

Several risks and challenges may be associated with RPM interventions, including overuse of digital monitoring and sustainability issues in patient care. Previous findings suggest that over-monitoring can be associated with psychological, physical, and other systemic risks, which may entail staff stress, reduced performance, and turnover [34]. Alarm fatigue, here, in health care, driven by excessive and often false alarms, as well as over-surveillance, may endanger staff mental health and patient safety [35]. Although these aspects were not examined in this, they merit particular attention and should be explored in future research. On the other hand, the long-term sustainability of RPM interventions, particularly in older adult patient care, can be a major challenge. We need to ensure their capacity to use resources wisely while maintaining high-quality care. To achieve this, it is essential to facilitate its adoption within health care systems, for example, through starting better reimbursement models that support their use and impact. Therefore, to help integrate the RPM system into older adult care on a larger scale, we suggest the following practical policy changes (Textbox 1).

Textbox 1. **Policy recommendations**

- Build on the EPOCA RPM system experience: use the success of initiatives like Article 51 to move from temporary trials to long-term funding for RPM systems.
- Develop specific reimbursement codes: define billing codes that cover the full RPM process, from the inclusion to daily monitoring and monthly reviews, so it can match how care is delivered in real life.
- Incentivize RPM systems through value-based contracts: this involves encouraging local health agencies to sign agreements with care providers that link payments to outcomes (eg, reduced hospital visits or better patient experiences), with bonuses being awarded for exceeding the benchmark.
- Ensure equitable access to RPM systems for diverse populations: this includes vulnerable older adults, especially those living in poverty or isolated areas.

### Study Limitations

Several limits must be acknowledged in this study. First, it is based on retrospective data, possibly leading to information gaps and potential biases, particularly regarding the data collected for the Y–1 and Y periods. To mitigate this, multiple measures have been taken to ensure comprehensive data collection, especially for the Y–1 period, using patient medical records and direct contact with their care team.

Second, the study sample size was relatively limited, as we only included patients with more than 1 year of follow-up to facilitate a more robust comparison between the 2 periods. This inclusion criterion was not considered in the first study, where patients with less than 1 year of follow-up were included in the analysis. Although the results were adjusted, this difference could potentially affect the comparability of the results in terms of different operators of the system (ie, geriatrician or GP).

Third, some factors such as differences in access to care, disparities in socioeconomic status, and adherence to RPM interventions were not specifically addressed in our analysis. Our primary focus was on measuring the impact on unplanned hospitalizations; we also provided secondary analysis related to patient characteristics, including age, sex, GIR score, comorbidity, and others. Looking forward, variables such as care accessibility, adherence to RPM, as well as cost and sustainability issues, could be further explored in future studies.

Fourth, this study covers the period between 2022 and 2024. It is important to acknowledge that following the COVID-19 pandemic, health care systems across Europe, including in France, have experienced an accelerated adoption of digital health solutions, such as remote monitoring systems. We believe that the observed effectiveness of the EPOCA RPM system in supporting the care management of complex older adult patients is reflecting the broader shifts in health care practices.

Finally, the absence of a specific control group, such as a usual or standard care group, which may not be feasible retrospectively, limits the generalizability of our findings. To address these limitations, a large-scale randomized controlled trial is set to begin in 2025 (NCT06845917) to generate more robust, high-level evidence.

### Conclusions

This study showed that, regardless of the system operator, the EPOCA RPM system can help in reducing unplanned hospitalization days and numbers, as well as ED visits, potentially alleviating hospitals’ burdens. These findings highlight the possibility of a new organization of at-home care for complex older adult patients and suggest RPM systems such as EPOCAs, as a potential alternative solution, especially in the context of limited geriatric resources. In perspective, further evidence regarding the effectiveness of our RPM system can be obtained through a large-scale randomized controlled trial.

## Supplementary material

10.2196/71527 Multimedia Appendix 1 Characteristics of the participants.

10.2196/71527 Multimedia Appendix 2 Additional analysis of outcome measures stratified by the number of hospitalizations in the Y-1 period, the disability severity (GIR score), and the nonprofessional caregiver status.

10.2196/71527 Multimedia Appendix 3 Comparison of characteristics of patients included in both studies.

## Abbreviations

ED: emergency department
GIR: Groupe Iso-Ressources (iso-ressources group)
GP: general practitioner
IADL: Instrumental Activities of Daily Living
RCT: randomized control trial
RPM: remote patient monitoring
