# Supplementary material for: Remote Patient Monitoring System for Polypathological Older Adults at High Risk for Hospitalization: Retrospective Cohort Study
Source: J Med Internet Res. 2025 Jul 14;27:e71527. doi: 10.2196/71527 (PMC12279313; doi:10.2196/71527)
Supplement: Multimedia Appendix 1 [file jmir-v27-e71527-s001.docx]

**Supplementary Material 1. Characteristics of the participants**

|  | N of missing data | All (n=80) |
| --- | --- | --- |
| **Demographic data** |  |  |
| Age (years), mean (SD) | 0 | 86.7 (8.3) |
| Sex (female), n (%) | 0 | 55 (69) |
| Social isolation, n (%) | 0 | 46 (58) |
| Presence of a non-professional caregiver, n (%) | 0 | 54 (68) |
| Number of children, mean (SD) | 2 | 1.9 (1.3) |
| **Professional caregiver** |  |  |
| Number of external caregivers, mean (SD) | 0 | 2.4 (1.7) |
| Nurse, n (%) | 4 | 33 (43) |
| Care assistant, n (%) | 3 | 36 (47) |
| Physiotherapist, n (%) | 3 | 22 (29) |
| Home nursing services, n (%) | 8 | 30 (42) |
| Home care helper, n (%) | 7 | 24 (32) |
| Housekeeper, n (%) | 9 | 31 (44) |
| Meal delivery, n (%) | 4 | 11 (14) |
| GP declared | 0 | 74 (92) |
| **Functional and cognitive assessment** |  |  |
| GIR, mean (SD) | 7 | 3.6 (1.5) |
| ADL - Katz index, mean (SD) | 12 | 4.0 (1.9) |
| IADL - Lawton index, mean (SD) | 18 | 3.8 (2.7) |
| **Nutritional assessment** |  |  |
| BMI, mean (SD) | 11 | 24 (5.0) |
| Undernutrition | 0 | 21 (28) |
| **Comorbidities** |  |  |
| Number of chronic diseases, mean (SD) | 0 | 6.7 (3.7) |
| Essential hypertension, n (%) | 0 | 50 (63) |
| Rhythm disturbance, n (%) | 0 | 23 (29) |
| Progressive ocular pathology, n (%) | 0 | 22 (28) |
| Abdominal syndrome, n (%) | 0 | 21 (26) |
| Bronchopulmonary disease, n (%) | 0 | 20 (25) |
| Dementia, n (%) | 0 | 20 (25) |
| Depression, n (%) | 0 | 19 (24) |
| Heart failure, n (%) | 0 | 19 (24) |
