# Supplementary material for: Remote Patient Monitoring System for Polypathological Older Adults at High Risk for Hospitalization: Retrospective Cohort Study
Source: J Med Internet Res. 2025 Jul 14;27:e71527. doi: 10.2196/71527 (PMC12279313; doi:10.2196/71527)
Supplement: Multimedia Appendix 2 [file jmir-v27-e71527-s002.docx]

**Supplementary Material 2:** Additional analysis of outcome measures stratified by the number of hospitalizations in the Y-1 period, the disability severity (GIR score) and the non-professional caregiver status

|  | **Number of unplanned hospitalizations days** | **Unplanned Hospitalizations** | **ED Visits** |
| --- | --- | --- | --- |
|  | **^a^RRR (%) /**  ***P* value** | **RRR (%) /**  ***P* value** | **RRR (%) /**  ***P* value** |
| **Number of Y-1 hospitalization** |  |  |  |
| Patients with no hospitalization prior year (N=20) | / 0.07 (NS) | / 0.03 | / 0.06 (NS) |
| Patients with 1 hospitalization prior year (N=44) | 44% / <.001 | 52% / <.001 | 65% / <.001 |
| Patients with 2 or more hospitalizations prior year (N=16) | 98% / <.001 | 86% / <.001 | 80% / 0.0019 |
| **Disability severity^b^** |  |  |  |
| No or moderate disability level (N=42) | 41% / 0.0016 | 55% / <.001 | 64% / <.001 |
| Severe disability level (N=31) | 56% / <.001 | 68% / 0.02 | 61% / 0.02 |
| **Presence of non-professional caregiver** |  |  |  |
| Presence of non-professional caregiver (N=54) | 41% / 0.0072 | 47% / 0.0048 | 59% / <.001 |
| Absence of non-professional caregiver (N=26) | 64% / <.001 | 78% / 0.0011 | 69% / 0.0018 |

**^a^_RRR (%) /_ *_P_* _value_**_:  refers to the change in Y-1 vs. Y and statistical significance between the two periods._

**^b^_Disability level:_**  _refers to the GIR evaluation. No or moderate disability level (GIR 4 - 5 or 6), and severe disability level (GIR 1 - 2 or 3)_
