# Supplementary material for: Remote Patient Monitoring System for Polypathological Older Adults at High Risk for Hospitalization: Retrospective Cohort Study
Source: J Med Internet Res. 2025 Jul 14;27:e71527. doi: 10.2196/71527 (PMC12279313; doi:10.2196/71527)
Supplement: Multimedia Appendix 3 [file jmir-v27-e71527-s003.docx]

**Supplementary Material 3.:** Comparison of characteristics of patients included in both studies

|  | **Total (N=80)** | **Testa et al. (2024) (N=120)** |
| --- | --- | --- |
| **Demographic characteristics** |  |  |
| Age (years), mean (SD) | 86.7 (8.3) | 86.8 (7.9) |
| Sex (female), n (%) | 55 (69%) | 71 (59%) |
| **Presence of caregiver, n (%)** |  |  |
| Non-professional caregiver, n (%) | 54 (68%) | 66 (55%) |
| Professional caregivers, mean (SD) | 2.4 (1.7) | 2.4 (1.7) |
| **Functional and cognitive assessment** |  |  |
| GIR^a^, mean (SD) | 3.6 (1.5) | 3.4 (1.3) |
| ADLs^b^ - Katz index, mean (SD) | 4.0 (1.9) | 3.6 (1.9) |
| IADL^c^ - Lawton index, mean (SD) | 3.8 (2.7) | 2.8 (2.3) |
| **Comorbidities** |  |  |
| Number of chronic diseases, mean (SD) | 6.7 (3.7) | 6.1 (2.5) |

^a^_GIR: Groupe Iso-Ressources (equal resource group)_

^b^ _ADL : Activities of Daily Living_

^c^ _IADL : Instrumental Activity of Daily Living_
